# Longitudinal Dietary Trajectories With Cognitive and Psychosocial Well-Being in Chinese Adults Aged 85 Years and Older in Singapore

**DOI:** 10.1093/geroni/igad036

**Published:** 2023-04-26

**Authors:** Jun S Lai, Grand H.-L Cheng, Yap-Seng Chong, Mary F.-F Chong, Woon-Puay Koh

**Affiliations:** Singapore Institute for Clinical Sciences, Agency for Science Technology and Research, Singapore, Singapore; Department of Medicine, Yong Loo Lin School of Medicine, National University of Singapore, Singapore, Singapore; Singapore Institute for Clinical Sciences, Agency for Science Technology and Research, Singapore, Singapore; Department of Obstetrics & Gynaecology, Yong Loo Lin School of Medicine, National University of Singapore, Singapore, Singapore; Singapore Institute for Clinical Sciences, Agency for Science Technology and Research, Singapore, Singapore; Saw Swee Hock School of Public Health, National University of Singapore and National University Health System, Singapore, Singapore; Singapore Institute for Clinical Sciences, Agency for Science Technology and Research, Singapore, Singapore; Healthy Longevity Translational Research Programme, Yong Loo Lin School of Medicine, National University of Singapore, Singapore, Singapore

**Keywords:** Cognitive impairment, Depressive symptoms, Diet quality, Longitudinal, Social engagement

## Abstract

**Background and Objectives:**

Studies on longitudinal trajectories of diet and the influence on aging in older adults are limited. We characterized diet quality trajectories over the past 2 decades among adults aged ≥85 years and examined their associations with cognitive and psychosocial outcomes.

**Research Design and Methods:**

We used data from 861 participants in the population-based Singapore Chinese Health Study. Dietary intakes were assessed at baseline (mean age [range]: 65 [60–74] years) and at follow-ups 3 (85 [81–95]) and 4 (88 [85–97]) years. Diet quality was measured by adherence to the Dietary Approaches to Stop Hypertension pattern, and group-based trajectory modeling was used to derive diet quality trajectories. At Follow-up 4, we assessed cognition using the Singapore-modified Mini-Mental State Examination, depressive symptoms using the 15-item Geriatric Depression Scale, social engagement, and self-rated health. Multivariable logistic regression models examined associations of diet quality trajectories with these outcomes.

**Results:**

About 49.7% had a trajectory with consistently low diet quality scores, whereas 50.3% had a trajectory with consistently high diet quality scores. Compared to the “consistently low” trajectory, the “consistently high” trajectory had 29% and 26% lower likelihoods of cognitive impairment and depressive symptoms, respectively (odds ratio, 95% confidence interval: 0.71 [0.51, 0.99] and 0.74 [0.55, 0.99], respectively); as well as 47% higher likelihood of social engagement (1.47 [1.09, 1.98]). No statistically significant association was observed between the trajectories and self-rated health.

**Discussion and Implications:**

Maintaining high diet quality throughout the older adult life course was associated with better cognitive and psychosocial well-being in adults aged ≥85 years.

**Translational Significance:** The link between a healthy diet and better cognitive and psychosocial well-being is well established, but longitudinal investigation on dietary changes over the past 2 decades among adults aged ≥85 years is scarce. We found that dietary patterns that are established earlier in life (ages 60–74 years) remain stable until very late life (85–97 years), and the maintenance of a high-quality diet was associated with better cognitive and psychosocial well-being, compared to the maintenance of a low-quality diet. Strategies to encourage the adoption of a high-quality diet need to start before older adulthood to improve cognitive and psychosocial well-being in very late life.

## Background and Objectives

Globally, the steady rise in life expectancy due to a decrease in later-life mortality has made the population of those aged ≥85 years the fastest-growing age group, and their numbers are expected to triple between 2020 and 2050 (World Health Organization [WHO], 2022). It is important to have high levels of cognitive and psychosocial well-being in older age such that individuals can continue to be independent and productive even in very late life (Halaweh et al., 2018). Individuals who survived beyond 85 years of age are a very heterogeneous population group, with considerable variability in cognitive function, mental health, and social engagement (Lowsky et al., 2014). However, studies that have investigated factors for survival or mortality among those aged ≥85 years often do not address the question of whether extra years of life gained through increased longevity in this population are spent in good or bad state of cognitive and psychosocial well-being (Lowsky et al., 2014).

Food is a key contributor to enjoyment, health, and well-being at all stages of the life course (Holder, 2019), and may influence the cognitive and psychosocial well-being in the oldest old. Studies in the oldest old showed that a favorable dietary pattern (e.g., lower meat intake, higher intakes of fruit and vegetables, whole grains, nuts and seeds, and lower intake of foods rich in saturated fats) was associated with better self-rated health (An et al., 2018), a lower risk of cognitive impairment or dementia (Granic et al., 2016; Nicoli et al., 2021; Yin et al., 2017), and depression (Granic et al., 2015), as well as greater psychological resilience (Yin et al., 2019). These aforementioned studies, however, are cross-sectional in design. A lifelong approach to higher levels of cognitive and psychosocial well-being is important as investments in health at younger ages (such as maintaining a healthy and active lifestyle) are profitable throughout life. Modeling dietary trajectories allows understanding of whether dietary patterns change over time and how a change in diet influences cognitive and psychosocial well-being in those aged ≥85 years, or whether the relation between diet and cognitive and psychosocial well-being reflects the accumulation of dietary exposures over time. Additionally, modeling dietary trajectories and classifying individuals into trajectory groups can provide important information about when and in whom to intervene. Although previous studies have explored dietary trajectories in children/adolescents (Appannah et al., 2020; Dalrymple et al., 2022) and adults (Batis et al., 2014; Winpenny et al., 2020), this remains an emerging area of nutritional epidemiology. To the best of our knowledge, only one study in a U.S. cohort has derived diet quality trajectories of older adults, and showed that those belonging to the “greatly improved” trajectory group had better physical function compared to those in the “moderately improved” trajectory group (Talegawkar et al., 2020).

Studies in Asian populations are critical as dietary patterns differ across cultures and countries (Luo et al., 2020). We have previously shown that maintaining adherence to a high diet quality (defined as above median diet quality scores at two time points) from mid- to late life (aged ≥45–≥65 years) was associated with a lower risk of cognitive impairment (Tong et al., 2021) and a higher likelihood of healthy aging (Zhou et al., 2022) in late life in a cohort of Chinese men and women in Singapore. This study will add on to previous evidence by (a) deriving diet quality trajectories using dietary intakes from three time points spanning from ages 60 to 74 years (late life) to ages 85 to 97 years (very late life), and (b) examining the associations between these trajectories and cognitive impairment, depressive symptoms, social engagement, and self-rated health among those aged ≥85 years of this cohort.

## Research Design and Methods

### Study Sample

The Singapore Chinese Health Study (SCHS) is an ongoing prospective cohort study in Singapore that recruited 63,257 Chinese adults aged 45–74 years between April 1993 and December 1998 (Hankin et al., 2001). Study participants were permanent residents or citizens who resided in government-built housing estates (where 86% of all Singaporeans resided during the recruitment period). Detailed descriptions of the SCHS have been published (Hankin et al., 2001). At baseline, in-person interviews were carried out to assess participants’ habitual diet, demographic factors, anthropometric measurements, lifestyle factors, and medical history. The participants were re-contacted for follow-up interviews in 1999–2004 (Follow-up 1), 2006–2010 (Follow-up 2), and 2014–2016 (Follow-up 3). All procedures in the SCHS were approved by the Institutional Review Board at the National University of Singapore, and written informed consent was obtained from all study participants.

From July 2017 to August 2018, we recruited the first 1,000 consenting SCHS participants who were 85 years and above, and who had participated in all previous follow-up studies in a special Follow-up 4 study called the SG90 Study (Chua et al., 2021). Participants attended in-person interviews that assessed aging-related outcomes such as cognitive function, quality of life, and functional independence, as well as lifestyle factors (including dietary intake), sociodemographic factors, anthropometry, and history of physician-diagnosed diseases. Further details of SG90 have been described elsewhere (Chua et al., 2021).

The current analysis included participants of the Follow-up 4 study (SG90) with dietary data at baseline, and Follow-ups 3 and 4 (SG90), as well as with complete data on cognitive impairment, depressive symptoms, social engagement, and self-rated health. This subset of participants was on average (mean) 65 years old at baseline (range: 60–74 years), 85 years old at Follow-up 3 (range: 81–95 years), and 88 years old at Follow-up 4 (range: 85–97 years).

### Assessments of Dietary Intake

Dietary intake at baseline was assessed with a validated 165-item, semiquantitative food frequency questionnaire (FFQ; Hankin et al., 2001), which was administered by trained interviewers. For each FFQ item, participants indicated their frequency of intake in the past 1 year from eight frequency options ranging from “never or hardly ever” to “2 or more times a day,” and the usual amount consumed from three portion-size images. For nonalcoholic beverages, participants indicated their consumption frequency of a standard serving (one glass/cup) from nine frequency options ranging from “never or hardly ever” to “6 or more times a day.”

Dietary intakes at SCHS Follow-ups 3 and 4 (SG90) were assessed with a dietary screener, which had good reproducibility and relative validity compared with a 163-item long FFQ in assessing a priori diet quality indices (i.e., the Alternative Healthy Eating Index-2010 [AHEI-2010], alternate Mediterranean Diet [aMed], and Dietary Approaches to Stop Hypertension [DASH] diet) in the local population as well as intakes of locally unique foods such as soy products and wholegrain rice and noodles (Whitton et al., 2018). The dietary screener was interviewer administered to assess the consumption of 21 food and beverage items in the past 1 year (Tong et al., 2021). Participants were asked to report their frequency of consuming one standard serving of each item ranging from “never or rarely” to “6 or more a day” (10 frequency options). Additional questions on the brand and name of breakfast cereals consumed were asked to help determine the whole grain content of the breakfast cereals.

### Assessment of Diet Quality

The method for assessment of diet quality at baseline and Follow-ups 3 and 4 (SG90) was adapted from the DASH diet (Fung et al., 2008). In a previous publication examining diet quality and healthy aging (including cognitive function, depressive symptoms, and self-rated health) in the same cohort (Zhou et al., 2021), similar associations were observed across different diet quality indices (i.e., DASH, AHEI-2010, aMED); hence, in the present study, we chose to represent overall diet quality using only the DASH score for ease of scoring.

The original DASH score included eight components and each component was assigned one to five points according to participant’s quintile of intakes. Participants in the highest quintile of five desired foods (whole grains, vegetables, fruits, nuts and legumes, and low-fat dairy) received five points, whereas three undesired foods (sugar-sweetened beverages and fruit juice, red and processed meat, and sodium) were reversely scored.

For the present study, we used total dairy intake (full- and low fat) as a surrogate for the low-fat dairy component of the original DASH score, due to lack of information on the latter (Tong et al., 2021). In SCHS, information collected on dairy products at all time points did not distinguish between full- or low fat. Additionally, we excluded the sodium component from the calculation of the total DASH score at all time points to ensure comparability because sodium intake was not adequately captured by the dietary screener used in Follow-ups 3 and 4 (Whitton et al., 2018). Thus, our modified DASH score consisted of seven components, excluding the sodium component, and had a maximum score of 35 points. All other components were scored the same as the original DASH score. Detailed methods on DASH scoring have been described elsewhere (Tong et al., 2021).

### Assessment of Cognitive and Psychosocial Outcomes

The outcomes examined in this study included cognitive function, depressive symptoms, social engagement, and self-rated health, which were captured at Follow-up 4 (SG90) visit. Cognitive function was evaluated using the Singapore-modified Mini-Mental State Examination (SM-MMSE; Feng et al., 2012), with cognitive impairment defined using the following education-level specific cutoffs: 17/18, 20/21, and 24/25 for those with no formal education, primary school education, and secondary school or higher education, respectively (Feng et al., 2012; Katzman et al., 1988). Further details of the SM-MMSE and its validation in the Singapore population can be found elsewhere (Feng et al., 2012). Depressive symptoms were assessed using the 15-item Geriatric Depression Scale (GDS-15; Sheikh & Yesavage, 1986), which had been validated in older Asians (Nyunt et al., 2009). Participants with a GDS-15 score of ≥5 were considered as having depressive symptoms. Social engagement was assessed with a question asking about the frequency of participating in social activities, senior club events, and/or attended a place of worship in the past month. The response options were “<1 per month,” “≥1 per month but <1 per week,” and “≥1 per week.” The latter two options were combined into the “≥1 per month” category for analysis. Self-rated health is a subjective measure of one’s own perceived health status, and was assessed in this study using one question “In general, would you say your health is: excellent, very good, good, fair, or poor?” This measure has been shown to be closely linked to a number of objective health indicators (Baron-Epel & Anderson, 2004). The responses were categorized into “poor or fair” or “good, very good, or excellent” for analysis.

### Assessment of Covariates

At baseline, in-person interviews were performed by trained research staff using a standardized questionnaire to obtain self-reported information on age, sex, highest educational level attained (no formal education, primary school, secondary school and above), marital status (married, separated/divorced, widowed, never married), cigarette smoking (never, former, current), alcohol drinking (none/occasional, weekly, daily), weekly physical activities (hours per week spent on moderate activities, strenuous sports, and vigorous work; categorized as none, 0.5–3.9, and ≥4.0 hr/week), sleep duration (≤5, 6–7, ≥8 hr/day). Body mass index (BMI; kg/m^2^) was based on self-reported weight and height, calculated as weight in kilograms divided by height in meters squared, then categorized according to World Health Organization (WHO) recommendations for weight categories in Asians: <18.5 underweight, 18.5–22.9 normal weight, ≥23.0–27.4 overweight or obese (WHO Expert Consultation, 2004).

Using the same questions, information on sleep duration was re-assessed at Follow-up 3, whereas marital status, cigarette smoking, and alcohol drinking were re-assessed at Follow-up 4 (SG90). Height and weight were based on self-reported data or measured by trained research staff at Follow-up 4 (SG90). Height was measured to the nearest 1 cm using a stiff, self-retracting, metallic tape measure, and weight was measured to the nearest 0.1 kg with a Soehnle Exacta Comfort digital weighing scale (Model S63315 PSD).

### Statistical Analysis

The DASH scores at all three time points (baseline, Follow-ups 3, and 4) were used in group-based trajectory modeling (GBTM), also known as Latent Class Growth Analysis, to identify latent classes of diet quality trajectories (Mplus version 8.8). This modeling method identifies groups of individuals following similar trajectories of a single variable within a study population (Jung & Wickrama, 2008). Several criteria were considered for selecting a trajectory model (class solution; Wickrama et al., 2021), and these included Akaike Information Criteria (AIC), Bayesian Information Criteria (BIC), and sample-size adjusted BIC (ssaBIC) in which lower values suggested better model fit. Classification accuracy was reflected by entropy, with values of 0.80, 0.60, and 0.40 representing high, moderate, and low accuracy, respectively. The Lo–Mendell–Rubin test was used to evaluate whether a model with *k* classes was significantly better (*p* < .05) than the counterpart with *k* − 1 classes. Following a rule of thumb, a model in which the smallest class involved at least 5% of the study sample is preferred. Moreover, considering the parsimony principle, linear trajectories were preferred over their nonlinear counterparts (Frankfurt et al., 2016; Jung & Wickrama, 2008).

Baseline characteristics of participants according to diet quality trajectories were compared by using chi-square test for differences in categorical variables and *t* test for differences in continuous variables. Multivariable logistic regression models were used to examine the associations of diet quality trajectories with each outcome. The models adjusted for age, year of baseline interview (1993–1995, 1996–1998) as the long recruitment period resulted in a large variation in follow-up intervals, sex, highest educational level, physical activity at baseline, as well as changes in the following: marital status (maintained married, maintained separated/divorced/widowed/never married, married to separated/divorced/widowed, and vice versa), cigarette smoking (maintained never/former, maintained current, current to never/former, and vice versa), alcohol drinking (maintained none/occasional, maintained weekly/daily, weekly/daily to none/occasional, and vice versa), sleep duration (maintained 6–7 hr, maintained ≤5/≥8 hr, 6–7 to ≤5/≥8 hr, ≤5/≥8 to 6–7 hr), and BMI status (maintained normal, maintained under/overweight and obese, normal to under/overweight and obese, under/overweight, and obese to normal). All analyses were performed using Stata version 17 (StataCorp LP, College Station, TX). We considered two-sided *p* < .05 to be statistically significant.

## Results

**Figure 1.**
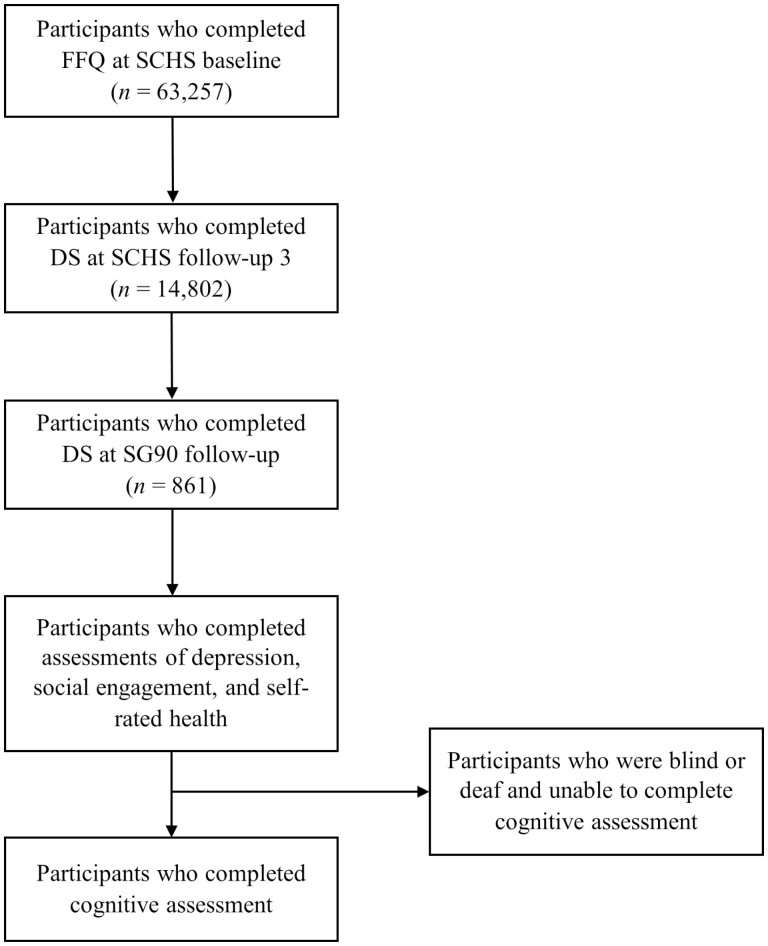
Participant flowchart. DS = Dietary Screener; FFQ = Food Frequency Questionnaire; SCHS =Singapore Chinese Health Study.

A total of 861 participants had dietary data at SCHS baseline, Follow-ups 3, and 4. All the participants completed assessments of depressive symptoms, social engagement, and self-rated health at Follow-up 4. A total of 11 participants were blind or deaf and could not complete the SM-MMSE, thus the analysis relating diet quality trajectories to cognitive impairment only included 850 participants (Figure 1).

**Figure 2.**
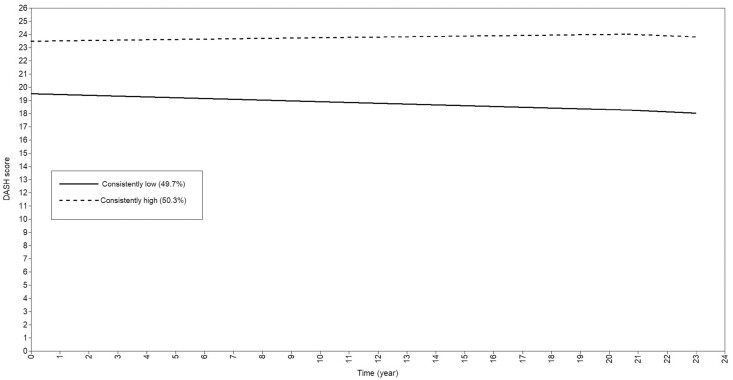
Diet quality trajectories over an average of 23 years, using group-based trajectory modeling, in a cohort of Chinese adults aged ≥85 years in Singapore.

A two-class linear model was identified as the optimal model based on model fit criteria. Results of model fit statistics for Classes 2–4 are presented in Supplementary Table 1. The two-class model had a moderate entropy (0.66), and its smallest class comprised 49.7% of the study sample. Although the AIC, BIC, and ssaBIC of the three- and four-class linear models were comparable to the two-class linear model, the three-class model had a lower entropy (0.55), and was not significantly better than the two-class model (*p* = .31), whereas the four-class model had only 4.3% of the study sample assigned to the smallest class. Models of quadratic trajectories were excluded as the quadratic terms in the two-class quadratic model were not statistically significant (*p* ≥ 0.17). In the selected two-class linear model (Figure 2), approximately 49.7% (*n* = 428) of participants was assigned to the trajectory characterized by a lower diet quality at baseline (intercept = 19.53, *p* < .001) which declined over time (slope = −0.06, *p* < .001); however, the decline was rather modest (<2 DASH score difference from baseline to Follow-up 4), thus we labeled this trajectory group as “consistently low.” The remaining participants (50.3%, *n* = 433) followed a trajectory characterized by a higher diet quality at baseline (intercept = 23.51, *p* < .001) and a nonstatistically significant slope (0.02, *p* = .080), thus labeled as the “consistently high” trajectory group. Individual trajectories with their corresponding DASH scores at each time point are shown in Supplementary Figure 1.

Participants in the “consistently low” trajectory group had an average (mean ± standard devaition) DASH score of 18 or 19 points at each time point, whereas participants in the “consistently high” trajectory group maintained their DASH scores at an average of 24 points at all three time points (Table 1). Participants in the “consistently low” trajectory group were more likely to have the lowest educational level, to be smoking, and to have the lowest physical activity level at baseline.

**Table 1.** Characteristics of Study Participants at Baseline (unless otherwise specified) by Diet Quality^a^ Trajectory Groups in a Cohort of Chinese Adults Aged ≥85 Years in Singapore

Characteristics	Consistently low (*n* = 428)		Consistently high (*n* = 433)		*p* ^b^
	Mean ± *SD*	*n* (%)	Mean ± SD	*n* (%)	
DASH score					
SCHS baseline	19.4 ± 3.6		23.7 ± 3.8		<.001
SCHS follow-up 3	18.1 ± 3.1		24.2 ± 3.1		<.001
SG90 follow-up	17.8 ± 3.2		24.0 ± 3.3		<.001
Age	64.6 ± 2.9		65.0 ± 3.1		.050
Male		163 (38.1)		141 (32.6)	.090
Highest education					.006
None		195 (45.6)		156 (36.0)	
Primary		185 (43.2)		204 (47.1)	
≥ Secondary		48 (11.2)		73 (16.9)	
Married		329 (76.9)		330 (76.2)	.760
Currently smoking		52 (12.2)		22 (5.1)	.001
Weekly alcohol drinking		25 (5.8)		27 (6.2)	.355
Physical activity					<.001
<0.5 hr/week		311 (72.7)		253 (58.4)	
0.5–3.9 hr/week		67 (15.6)		104 (24.0)	
4–6 hr/week		50 (11.7)		76 (17.6)	
Sleep					.143
≤5 hr/day		41 (9.6)		60 (13.9)	
6-7 hours/day		257 (60.0)		244 (56.3)	
≥8 hr/day		130 (30.4)		129 (29.8)	
BMI status					.101
Normal		152 (35.5)		182 (42.0)	
Underweight		39 (9.1)		25 (5.8)	
Overweight/obese		237 (55.4)		226 (52.2)	

*Notes*: BMI = body mass index; DASH = Dietary Approaches to Stop Hypertension; SCHS = The Singapore Chinese Health Study; *SD* = standard deviation; SG90 = a special Follow-up 4 study of the original SCHS.

^a^Defined by DASH score.

^b^ *p* Values are for *t* test or chi-square test of differences between groups.

When we examined the associations of diet quality trajectories with cognitive and psychosocial outcomes, after adjusting for confounders, we found that compared to those in the “consistently low” trajectory group, participants in the “consistently high” trajectory group had 29% (odds ratio [OR]: 0.71, 95% confidence interval [CI]: 0.51, 0.99) lower odds of cognitive impairment, 26% (OR: 0.74, 95% CI: 0.55, 0.99) lower odds of depressive symptoms, and 47% (OR: 1.47, 95% CI: 1.09, 1.98) higher likelihood of social engagement (Table 2). There were no statistically significant association between diet quality trajectories and self-rated health.

**Table 2.** Associations of Diet Quality Trajectories With Cognitive Impairment, Depression, Social Engagement, and Self-Rated Health in a Cohort of Chinese Adults Aged ≥85 Years in Singapore

Variable	Consistently low		Consistently high				
	Case/*n*	OR (95% CI)	Case/*n*	Unadjusted		Adjusted^a^	
				OR (95% CI)	*p*	OR (95% CI)	*p*
Cognitive impairment	116/423	1.00	100/427	0.81 (0.59, 1.10)	.180	0.71 (0.51, 0.99)	.041
Depression	172/428	1.00	146/433	0.76 (0.57, 1.00)	.049	0.74 (0.55, 0.99)	.042
Social Engagement	124/428	1.00	171/433	1.60 (1.20, 2.13)	.001	1.47 (1.09, 1.98)	.012
Self-rated health	128/428	1.00	153/433	1.28 (0.96, 1.70)	.090	1.28 (0.95, 1.71)	.108

*Notes*: BMI = body mass index; CI = confidence interval; OR = odds ratio.

^a^ Models adjusted for age, year of baseline interview (1993–1995, 1996–1998), sex, highest educational level and physical activity at baseline, and changes in the following: marital status, current smoking status, weekly alcohol drinking, sleep duration, and BMI status.

## Discussion and Implications

In this cohort of Singaporean Chinese adults aged ≥85 years, we found two diet quality trajectories spanning late to very late life, namely the “consistently low” trajectory which is characterized by low DASH scores at baseline and a modest decline over time, and the “consistently high” trajectory which is characterized by high DASH scores at baseline which maintained over time. Participants in the “consistently high” trajectory were found to have lower risks of cognitive impairment and depressive symptoms, as well as a higher likelihood of social engagement in very late life, compared to those in the “consistently low” trajectory.

This is the first study, to the best of our knowledge, that has applied the GBTM approach to longitudinal dietary data collected at three time points from late to very late life, and explored associations of diet quality trajectories with cognitive and psychosocial outcomes. In addition, the present study focused on those aged ≥85 years, which is a population subgroup underrepresented in aging studies. Our study findings are supported by the extensive literature on the beneficial role of a healthy diet in several aging outcomes. Whether in those aged 65–84 years or those aged ≥85 years, studies have shown that a healthy dietary pattern (defined either by dietary indices/scores, e.g., Mediterranean diet score, or established through statistical techniques, e.g., factor analysis) was associated with better physical function (Coelho-Junior et al., 2021), better self-rated health/quality of life (Govindaraju et al., 2018), lower risks of cognitive decline (Chen et al., 2019; Wu et al., 2019) and depression (Wu et al., 2020), and a higher likelihood of healthy aging (Foscolou et al., 2019; Zhou et al., 2021). However, only limited studies on longitudinal diet have focused on these outcomes in individuals aged ≥85 years. Previously in the SCHS cohort, we have taken an a priori approach to categorize participants into groups of changes in diet quality scores, and showed that those with consistently high diet quality from mid- to late life had a lower risk of cognitive impairment (Tong et al., 2021) and a higher likelihood of healthy aging (Zhou et al., 2022). However, such categorization is subjectively defined and does not capture the distinct diet trajectories presently occurring in the population understudied (Nagin & Odgers, 2010). Moreover, analyses of change in scores have been criticized for providing misleading effect estimates (Tennant et al., 2021). More recently, several modeling approaches have been developed to model longitudinal diet within older populations but tended to focus on average trajectories over time (An et al., 2018; Berendsen et al., 2017; McEvoy et al., 2019; Yan et al., 2022; Zhu et al., 2015). In the present study, we have advanced these prior analyses by using the GBTM approach to compute diet quality trajectories. This approach describes the variation in diet quality over time within the cohort, which offers new insights into identifying time points and subgroups for dietary interventions, as well as avoids the limitations associated with change-score analyses (Tennant et al., 2021).

The trajectories identified in this study suggest that dietary behaviors which are established earlier in life remain fairly consistent in the long term until very late in life. Those who started with a high diet quality at late life tended to maintain a high-quality diet until very late life, which were associated with better cognitive and psychosocial well-being in this cohort of Chinese adults aged ≥85 years (evident by lower risks of cognitive impairment and depression, and higher likelihood of social engagement). This finding provides additional evidence to the growing awareness within the public health communities on the importance of a life-course approach to health (Kuh et al., 2003). To achieve optimal cognitive and psychosocial well-being in very late life, public health interventions need to start in the decades leading up to very late life. Policy measures that support the adoption of a high-quality diet at early time points of adulthood could have continued benefits across the adult life course and impact on cognitive and psychosocial well-being in very late life. Nevertheless, our findings need to be interpreted with caution in the absence of baseline cognitive and psychosocial outcomes to rule out reverse causality, as it is possible that those with consistently high diet quality over time already had lower prevalence of cognitive impairment and depressive symptoms, and higher prevalence of social engagement at baseline, than those with consistently low diet quality.

Approximately half of the study sample had a low-quality diet from late to very late life. Although these individuals survived until very late life, their poor diet over time was associated with increased likelihood of cognitive impairment, depression, and social isolation. This is concerning and underscores the need for more interventions and health promotion efforts to support these individuals to adopt a healthier diet as they transition from late to very late life. Consistent with existing evidence showing clustering of unhealthy lifestyle behaviors (Mawditt et al., 2019), we found that those who consistently have a low-quality diet were also more likely to have other risk factors of poor health outcomes such as smoking and low physical activity level at baseline (Fisher et al., 2011). This finding again suggests the importance of early interventions to lifestyle behavior, and highlights a potential need for interventions to target multiple lifestyle behaviors in parallel for effective promotion of optimal cognition and psychosocial well-being. Nevertheless, this finding requires confirmation in future studies specifically designed to address clustering of unhealthy behaviors and the impact on cognitive and psychosocial well-being in individuals aged ≥85 years.

The strengths of our study include the longitudinal study design with repeated measurements of diet and important covariates, and the long follow-up time of 23 years leading up to very late life. In addition, the present study focused on those aged ≥85 years, which is a population subgroup that has not been widely studied. Our study is also novel in that we considered multiple aspects of health including mental, cognitive, and social outcomes which provides a holistic and comprehensive understanding of the influence of diet on health at very late life. Several limitations should be noted. As aforementioned, reverse causation is possible as we did not measure baseline cognition, depressive symptoms, and social engagement. The dietary questionnaire used at baseline differs from those at subsequent follow-ups, as such, diet quality across the time points may not be directly comparable, although the same food items were included for the computation of the DASH scores. We did not adjust for a change in physical activity level because different assessment methods were used for measuring of physical activity level at SCHS baseline and at SG90 follow-up. Likewise, a change in sleep duration was only between baseline and Follow-up 3, but we do not expect sleep duration to differ significantly between the follow-ups as the assessment period was only a difference of 3 years. The effect estimates may be affected by survivorship bias. If the relationship between diet and cognitive impairment, depression, and social engagement were weaker (stronger) among those who did not survive, we would overestimate (underestimate) the diet effects. Nevertheless, in this group of highly selective individuals, we still observed differences in associations between those with a consistently high-quality diet versus those with a consistently low-quality diet.

In summary, with a globally aging population, it is imperative to understand the factors contributing to higher levels of cognitive and psychosocial well-being in very late life. This study found that maintaining a high-quality diet from late to very late life was associated with lower risks of cognitive impairment and depression as well as a higher likelihood of social engagement. These findings support continuous efforts in advocating a high-quality diet throughout the life course of older adulthood, or even earlier in life, for cognitive and psychosocial well-being in very late life, although further studies are required to confirm temporality of this relationship.

## Supplementary Material

igad036_suppl_Supplementary_Materials

## Data Availability

The authors agree to provide the data, code book or analytic code used in the present analysis to the editors upon request. The SCHS-SG90 study is registered at clinicaltrials.gov as NCT03356340.
