# Supplementary material for: Longitudinal Dietary Trajectories With Cognitive and Psychosocial Well-Being in Chinese Adults Aged 85 Years and Older in Singapore
Source: Innov Aging. 2023 Apr 26;7(4):igad036. doi: 10.1093/geroni/igad036 (PMC10205470; doi:10.1093/geroni/igad036)
Supplement: igad036_suppl_Supplementary_Materials [file igad036_suppl_supplementary_materials.docx]

**Online Supplementary Material**

**Supplementary Table 1**: Model fit statistics of group-based trajectory modelling (linear trajectories) for diet quality in a cohort of Chinese adults aged 85 years and older in Singapore

|  | Models | | |
| --- | --- | --- | --- |
|  | 2-class | 3-class | 4-class |
| AIC | 14560.58 | 14527.62 | 14519.50 |
| BIC | 14598.65 | 14579.96 | 14586.12 |
| ssaBIC | 14573.24 | 14545.02 | 14541.65 |
| Entropy | 0.66 | 0.55 | 0.62 |
| Smallest class (%) | 428 (49.7%) | 217 (25.2%) | 37 (4.3%) |
| *P* (LMR) | < 0.001 | 0.31 | 0.24 |
| Notes. AIC = Akaike Information Criteria, BIC = Bayesian Information Criteria; ssaBIC = sample-size adjusted BIC; *P* (LMR) = *P*-value of Lo-Mendell-Rubin test. | | | |


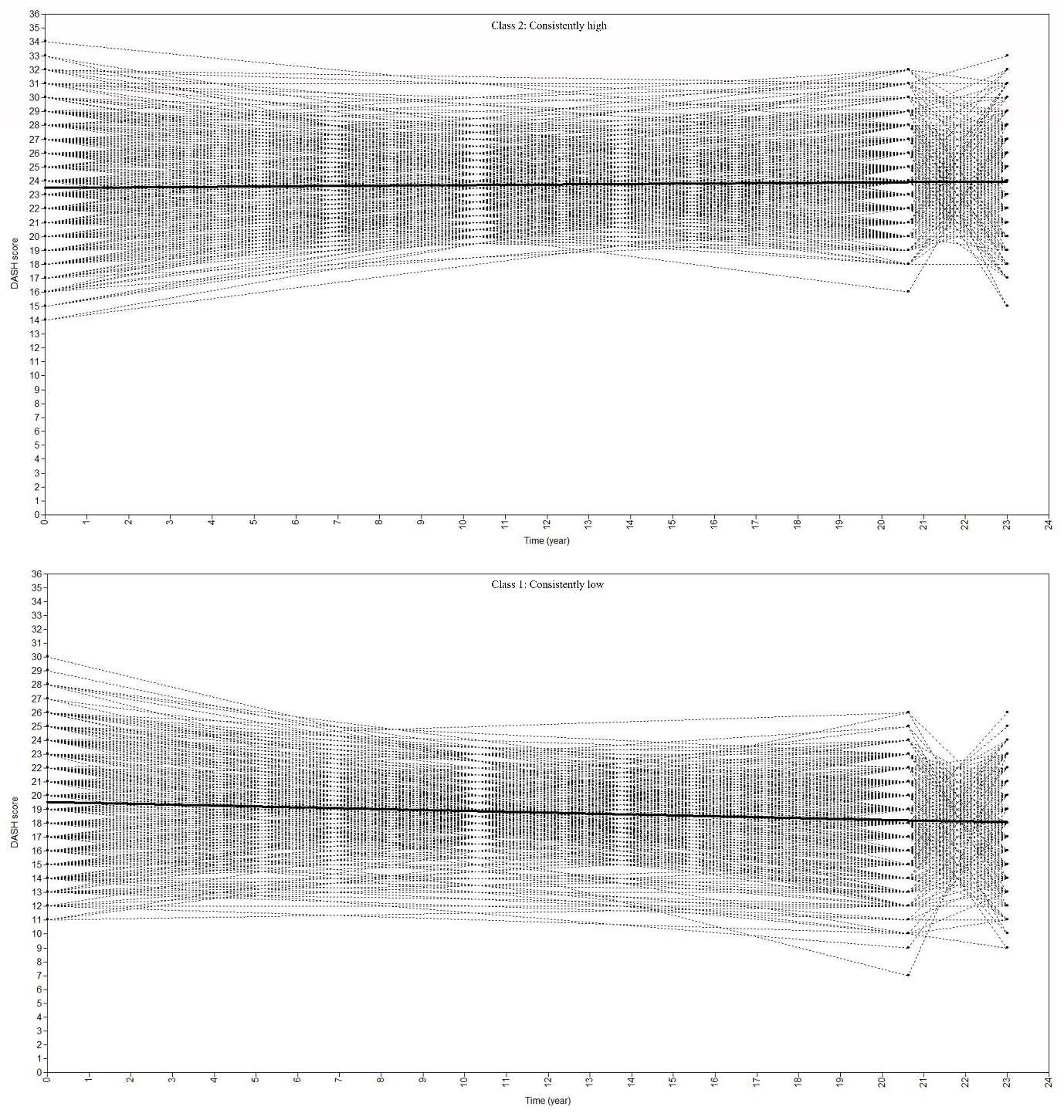


**Supplementary Figure 1**: Individual trajectories and DASH scores at each time point for the 2-class group-based trajectory model
